# Social determinants of health on attitudes toward childbearing among women with multiple sclerosis: A cross‐sectional study

**DOI:** 10.1002/brb3.70031

**Published:** 2024-09-12

**Authors:** Nahid Abbasi Khoshsirat, Romina Mokaram, Zohreh Mahmoodi, Ehsan Shahrestanaki, Nooshin Ghavidel

**Affiliations:** ^1^ Department of Neurology Shahid Rajaei Clinical Research and Development Unit Alborz University of Medical Sciences Karaj Iran; ^2^ School of Medicine Zhejiang University Hangzhou China; ^3^ Social Determinants of Health Research Center Alborz University of Medical Sciences Karaj Iran; ^4^ Non‐Communicable Diseases Research Center Alborz University of Medical Sciences Karaj Iran

**Keywords:** attitude, childbearing, depression, mental health, multiple sclerosis, social support

## Abstract

**Background:**

Pregnancy and motherhood are very valuable but challenging for women with multiple sclerosis (MS). Given that there are limited studies in this field, this study aimed to determine the social determinants of health on attitudes toward childbearing among women with MS.

**Methods:**

We conducted a cross‐sectional study on 206 women with MS in Alborz province, Iran, from February to June 2023 using convenience sampling. The data were collected using the questionnaire, and a linear regression analysis was applied.

**Results:**

The mean age of the participants was 36.80 ± 6.50 years. Participants’ Attitudes toward Fertility and Childbearing (PAFC) had a positive significant association with social support (*B* = .10, SE = .04, *p* = .023) and a significant negative association with the total score of depression, anxiety, and stress scale (*B* = −.13, SE = .06, *p* = .047) and depression (*B* = −.40, SE = .18, *p* = .023). However, the association between anxiety (*B* = −.25, SE = .20, *p* = .211), stress (*B* = −.36, SE = .18, *p* = .050), MS severity (*B* = .04, SE = .30, *p* = .890), and socioeconomic status scale (SES) (*B* = −.08, SE = .32, *p* = .806) was nonsignificant with PAFC.

**Conclusion:**

Our results showed that factors, including social support and mental health especially depression, can affect PAFC in women with MS. Therefore, it is necessary to determine specific strategies for policymakers to help MS patients manage pregnancy and motherhood.

## Graphical abstract

The Attitudes toward Childbearing among Women with Multiple Sclerosis is better in people with higher social support and less depression.

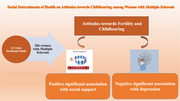

## 1 INTRODUCTION

Multiple sclerosis (MS) is an inflammatory disease with immune‐mediated myelin destruction of the central nervous system that has diverse presentations and an unclear pathogenesis (Lemus et al., 2018; McDonald et al., 2001). This disease has affected 2.5 million people all over the world, and its incidence is increasing. Studies have shown that various causes such as genetics, environment, and geographical location play a role in the incidence of this disease, so different communities with their unique social and individual characteristics have led to differences in the distribution of MS (Rosati, 2001). In Iran, the incidence and prevalence rates of MS have been increasing since 1990 (Fattahi et al., 2021).

MS is usually diagnosed between the ages of 20 and 40, when people are most likely to have children (Azami et al., 2019; Etemadifar et al., 2013). MS affects young women 3–1 more than men, and as such, pregnancy concerns are important for MS patients and their families (Alwan & Sadovnick, 2012). Pregnancy and motherhood are valuable for women with MS, but they face serious challenges related to childbearing. They worry about pregnancy complications, possible infertility, the lack of social support, receiving conflicting or wrong information, and the negative effects of the disease on their physical abilities. These concerns lead to postponing pregnancy, losing time to have children, and experiencing motherhood. Therefore, it is necessary to determine specific strategies for managing pregnancy and motherhood (Ghafoori et al., 2020; Kosmala‐Anderson & Wallace, 2013).

Studies have shown that MS does not have an adverse effect on the outcome of pregnancy. MS relapse rates typically decrease by 70% in late pregnancy but increase in the postpartum period. The reasons for the increase in activity after delivery are not completely clear, but factors such as the sudden drop in estrogen levels immediately after delivery and the loss of the immunosuppressive state of pregnancy are likely to be important (Arneth, 2022; Vukusic et al., 2021). Other studies have shown that pregnancy complications, including gestational diabetes and an increased risk of neonatal outcomes, such as preterm birth, low Apgar score, and congenital malformations were significantly more common in MS patients than in groups of healthy women. Therefore, this can cause concern in MS patients (Jesus‐Ribeiro et al., 2017; Rahmati et al., 2024).

Many factors, such as age, education, employment status, husband and wife's job status, attitude, mental health, hope, perceived social support, and marital satisfaction, are significant predictors of childbearing intention. Psychological factors such as marital satisfaction and social support have a great impact on the process of childbearing (Araban et al., 2020). Social support is an available interpersonal resource that can help patients with MS improve their quality of life (QoL) (Papa et al., 2021). Studies have shown that depression and anxiety are both common in MS and are associated with many outcomes. Depression and anxiety are associated with progression of disability in MS. Social support is a predictor of anxiety and depression (Hanna & Strober, 2020; Vorobeychik et al., 2020).

Qualitative studies of the childbearing experience of women with MS showed that limited access to information about the relationship between MS and childbearing, receiving incorrect information, opinions of family members and doctors about childbearing, trust and emotional support influenced the experience of women with MS in making decisions about childbearing (Fragkoudi et al., 2023; Kosmala‐Anderson & Wallace, 2013).

Given the above, pregnancy and motherhood are very valuable but challenging for women with MS. Considering that MS patients suffer from physical problems followed by mental problems, social support can be very important to reduce their problems. As there are limited studies in this field, the aim of this study was to determine the social determinants of health on attitudes toward childbearing in women with MS.

## 2 MATERIALS AND METHODS

### 2.1 Design and participants

This cross‐sectional study was conducted on 206 women with MS who had been referred to the MS Center of Shahid Rajaei Hospital, Karaj, Iran for routine neurological follow‐up, using convenience sampling from February to June 2023. The sample size was calculated with a Type I error of 0.05% and a power of 0.8, resulting in a sample size of 216. The inclusion criteria were patients with MS aged 18–48, absence of physical disability or mental illness before the diagnosis of MS, having enough literacy to answer the questions, and at least 6 months have passed since the final diagnosis of MS. Exclusion criteria were people who have severe limitations in activities that require the help of others or the use of other aids, history of brain damage such as tumor, and unwillingness to cooperate. After selecting the subjects, trained experts collected data by interview, and using a questionnaire. Informed consent was obtained from all participants after being informed of the nature of the research.

### 2.2 Data collection

Data were completed using a questionnaire, including

1. Sociodemographic checklist, including age, occupation, education, number of family members, and income. Questions about pregnancy and delivery include the age of marriage, number of pregnancies, delivery, abortion, breastfeeding, and children, as well as pregnancy complications, type of delivery, weight, and abnormalities of the baby. Information related to MS disease includes the type of disease, date of disease onset, underlying disease, family history of MS, and severity of MS disease during pregnancy and after delivery.
2. Socioeconomic status scale (SES): SES consisted of 6 questions including education, income, economic class, and housing status, which are scored based on a Likert scale from 1 to 5, and a total score ranging from 6 to 30. Validity and reliability have been performed in Iran (Eslami et al., 2014).
3. The MS Related Severity checklist (Bove et al., 2013) consisted of seven items. Patients rated their disability on a 0–4 scale in seven areas (walking, use of upper extremities, speech disturbance, vision, dysphagia, cognitive or affective disturbance, and sensory disturbance). The total score range is from 0 to 28. In our study, Cronbach's alpha on this checklist was .742.
4. Depression, anxiety, and stress scale (DASS)‐21 questionnaire (Lovibond & Lovibond, 1995): We used the Persian version of the DASS—21 Items (DASS‐21) for the assessment of depression, anxiety, and stress that is reliable and valid (Asghari et al., 2008). The DASS‐21 is a self‐report questionnaire consisting of 21 items, 7 items for each category of depression, anxiety, and stress. Patients are asked to score every item on a scale from 0 (did not apply to me) to 3 (applied to me very much). Total scores were calculated by summing the item scores in each subscale. Therefore, the total scores for the DASS‐21 scale ranged from 0 to 63.
5. Medical outcomes study (MOS) social support scale (MOS‐SSS) (Sherbourne & Stewart, 1991). MOS‐SSS was developed for patients in the MOS, a 2‐year study of patients with chronic conditions. This scale is a self‐reporting tool and measures the amount of social support received by the subject. This scale has 19 statements and 4 subscales including tangible support, emotional/informational support, positive social interaction, and affectionate support that are scored on a 5‐point Likert scale. To obtain the score for each subscale, the scores of the statements related to the subscale are added together. To get the overall score, all scores are added together, the lowest score in this test is 19 and the highest score is 95. The high score of the subject on this scale indicates good social support. The reliability and validity of this questionnaire were done in Iran (Mohammadzadeh et al., 2016).
6. Attitude toward fertility and childbearing (AFC) scale: This scale was first compiled by Söderberg et al. (2013) and is used to evaluate women's AFC. The reliability and validity of this questionnaire were done in Iran (Baezzat et al., 2017). This scale has 23 questions and 4 subscales, including children as the basis of life, the child as a barrier, postponing the fertility to future, and fertility after the fulfillment of preconditions. Participants were asked to assess the statements on a 5‐point Likert scale (1  =  very disagree to 5  =  very agree). A higher score indicates a more positive attitude toward fertility and childbearing.

### 2.3 Statistical analysis

All analyses were conducted using SPSS 22.0 statistical software. Descriptive statistics were used to summarize the demographic characteristics of the study population, including the calculation of means and standard deviation (SD) for continuous variables and frequency (%) for categorical variables. We employed a two‐tailed *t*‐test to compare mean AFC score with education and abortion as well as one‐way analysis of variance (ANOVA) for comparisons of AFC with family, pregnancy, and child numbers. Additionally, a linear regression analysis was performed to investigate the association between AFC and social support, mental health, anxiety, stress, depression, MS severity, and SES. A *p*‐value of less than .05 was considered statistically significant.

## 3 RESULTS

Overall, 206 women with MS participated in the survey. The mean age of the participants was 36.80 ± 6.50 years. The most common type of MS (81.3%) was relapsing–remitting MS. On average, the mean marriage age of women was 21.50 ± 4.05 years. The mean (SD) of AFC, social support, DASS, SES, and MS severity scores were 66.75 ± 13.85, 76.20 ± 21.30, 20.20 ± 14.10, 9.35 ± 3.00, and 2.90 ± 3.20, respectively. Table 1 presents more detailed information about the demographic and main characteristics of participants.

**TABLE 1:** Demographic and main characteristics of participants.

Variables		No. (%)	Variables	(Mean ± SD)
**Family member**	2	62 (30.10)	Age	36.80 ± 6.50
	3	66 (32.00)	Marriage age	21.50 ± 4.05
	≥4	77 (37.40)	Income, Million Rials	100.40 ± 40.40
**Pregnancy number**	0	58 (28.20)	Social support	76.20 ± 21.30
	1	55 (26.70)	SES	9.35 ± 3.00
	2	65 (31.60)	MS severity	2.90 ± 3.20
	3	19 (9.20)	Depression	5.80 ± 5.40
	≥4	9 (4.40)	Anxiety	5.80 ± 4.80
**Children number**	0	63 (30.60)	Stress	8.70 ± 5.20
	1	66 (32.00)	DASS	20.20 ± 14.10
	2	68 (33.00)	Pregnancy attitude	66.75 ± 13.85
	3	8 (3.90)		
**Abortion**	Yes	35 (17.00)		

Abbreviations: DASS, depression, anxiety, and stress scale; MS, multiple sclerosis; SD, standard deviation; SES, socioeconomic status scale.

In 25.00% of the subjects, pregnancy occurred after their diagnosis of MS, and 97.2% reported that they conceived naturally. Regarding pregnancy complications, they reported no complications (85.1%), gestational diabetes (5.4%), and preterm delivery (3.4%). All parents confirmed that their newborns were healthy, and 95.1% had breastfeeding. Overall, 97.9% of MS patients who experienced pregnancy reported that they had no disease attacks during pregnancy. Overall, 32.9% of women reported attacks of illness after delivery, with 57.5% attacks in the first year.

We examined the factors affecting participants’ AFC, including education, number of family members, number of pregnancies, number of children, and having abortion through *t*‐test and ANOVA analysis. However, none of these factors had a statistically significant effect on participants' AFC (*p*‐value >.05) (Table 2).

**TABLE 2:** Factors affecting participants’ attitude toward fertility and childbearing (AFC) based on *t*‐test and analysis of variance (ANOVA) analysis.

Variables		Mean ± SD	*p*‐Value
**Education**	Nonacademic	67.00 ± 14.60	*p*‐Value = .732^a^
	Academic	66.30 ± 12.40	
**Number of family members**	2	67.00 ± 15.40	*F* = .862^b^ *p*‐Value = .424
	3	68.40 ± 13.00	
	4 or more	65.40 ± 13.15	
**Number of pregnancies**	0	67.80 ± 15.05	*F* = .982^b^ *p*‐Value = .418
	1	68.50 ± 12.40	
	2	64.80 ± 13.50	
	3	63.60 ± 13.85	
	4 or more	69.90 ± 16.70	
**Number of children**	0	67.30 ± 14.90	*F* = .710^b^ *p*‐Value = .547
	1	68.20 ± 13.40	
	2	65.75 ± 12.70	
	3	61.90 ± 18.00	
**Abortion**	Yes	66.40 ± 14.70	*p*‐Value = .868^a^
	No	66.80 ± 13.70	

Abbreviation: SD, standard deviation.

^a^ *t*‐Test analysis.

^b^ One‐way ANOVA.

Table 3 shows a linear regression analysis investigating the association between participants’ AFC (PAFC) and variables of the study. The analysis included both unadjusted and adjusted models. Results of adjusted linear regression analysis showed that there is a positive significant association between social support (*B* = .10, SE = .04, *p* = .023) and PAFC, as well as there is a significant negative association between PAFC with DASS score (*B* = −.13, SE = .06, *p* = .047) and depression (*B* = −.40, SE = .18, *p* = .023). However, the association between anxiety (*B* = −.25, SE = .20, *p* = .211), stress (*B* = −.36, SE = .18, *p* = .050), MS severity (*B* = .04, SE = .30, *p* = .890), and SES (*B* = −.08, SE = .32, *p* = .806) was nonsignificant with PAFC.

**TABLE 3:** Association between the participants’ attitudes toward fertility and childbearing (PAFC) and variables of the study in the linear regression model.

Variables	Unadjusted		Adjusted^a^	
	*B* (SE)	*p*‐Value	*B* (SE)	*p*‐Value
**Social support**	.12 (.04)	.008	.10 (.04)	.023
**DASS**	−.14 (.07)	.032	−.13 (.06)	.047
**Anxiety**	−.27 (.20)	.171	−.25 (.20)	.211
**Stress**	−.40 (.18)	.032	−.36 (.18)	.050
**Depression**	−.43 (.18)	.016	−.40 (.18)	.023
**MS severity**	−.06 (.30)	.840	.04 (.30)	.890
**SES**	.05 (.32)	.863	−.08 (.32)	.806

Abbreviations: DASS, depression, anxiety, and stress scale; MS, multiple sclerosis; SES, socioeconomic status scale.

^a^ Adjusted for age.

## 4 DISCUSSION

Our study showed that the mean score of PAFC was less than normal women in Iran (Alijanzadeh et al., 2023). This may be due to the many worries of women with MS, including MS worsening with pregnancy, ability to care for child secondary to MS, passing MS onto child, stopping disease‐modifying therapies to attempt pregnancy, and lack of knowledge about options for pregnancy and MS (Kelly et al., 2024). In line with our study, Alwan and colleagues showed that 79.1% of respondents did not become pregnant following the diagnosis of MS. The most common reasons were concerns about inadequate parenting abilities, transmission of MS to the child, and possible risks associated with exposure to medications during pregnancy (Alwan et al., 2013).

According to the results of the present study, having social support had a positive significant association with PAFC. Good social support and relationships contribute significantly to health and strongly protect health. Social support can lead to healthier behaviors and predict QoL (Silveira et al., 2019). Ghafoori et al. (2020), in their qualitative study, stated that the majority of women with MS do not receive enough support from their families, society, and relevant organizations, which causes their concern about pregnancy. Rehabilitation treatment staff only focus on the physical problems of MS patients and do not pay attention to other aspects, including pregnancy. In line with our study, Sadeghi and Saraie (2016) found that people who have more support from parents, relatives, and friends, compared to people who have less social support have a greater desire to have children and fulfill their intentions. A study conducted on healthy women with no children or one child in Iran also showed that governmental childbearing incentives, generalized trust, and marital satisfaction had a positive and significant relationship with PAFC (Alijanzadeh et al., 2023).

In the present study, having mental disorders (total score of the DASS questionnaire) had a negative association with the PAFC, and in examining the dimensions measured by the mentioned instrument, only depression had a statistically significant negative relationship with the PAFC. Studies have shown that depression is highly prevalent in MS patients and is associated with the progression of disability in MS. Depression causes poorer treatment adherence and decreased QoL (Silveira et al., 2019; Vorobeychik et al., 2020). A systematic review and meta‐analysis conducted in 2017 on women with MS showed that depression has a negative impact on the QoL of these women (Boeschoten et al., 2017). QoL can have a direct relationship with the desire of families for fertility and childbearing (Chen et al., 2018). Moradi et al. (2021) also found that the increase of different dimensions of the QoL in women increases the desire for fertility and childbearing, especially dimensions of mental health and physical health.

In our study, there was an inverse relationship between MS severity and PAFC, but this relationship was not significant, which may be because the MS women in our study had mild disease severity. A qualitative study found that women with physical disabilities were worried that they would not be able to take care of their child or do activities with the child such as walking, running, or attending school meetings (Ghafoori et al., 2020). Studies have shown that in recent decades, there has been an increase in counseling women with MS and pregnancy. Women who treated before and during pregnancy with high‐efficacy disease‐modifying drugs had lower relapse rates before and after pregnancy (Kelly et al., 2024; Toscano et al., 2023).

Our study had several limitations: First, participants in this study were from a sample of MS patients in Karaj city, which makes the sample less representative of the general population of Iran. Other studies with samples from different areas are needed to elucidate factors involved in AFC among MS patients. Second, all the data in our study were self‐reported.

## 5 CONCLUSION

In this study, we evaluated the social determinants of health on attitudes toward childbearing in women with MS. Results showed that PAFC had a significant positive association with social support and a significant negative association with DASS score and depression. There was a nonsignificant association between anxiety, stress, MS severity, and SES with PAFC. Considering that women with MS suffer from physical problems followed by mental problems, social support may help them overcome the challenges of pregnancy and motherhood. Therefore, it is necessary to determine specific strategies for policymakers to help MS patients manage pregnancy and motherhood, such as increasing social support and mental health.

## AUTHOR CONTRIBUTIONS

**Nahid Abbasi Khoshsirat**: Conceptualization; writing—original draft; writing—review and editing; supervision. **Romina Mokaram**: Data curation; writing—original draft; writing—review and editing. **Zohreh Mahmoodi**: Formal analysis; writing—original draft; writing—review and editing; methodology. **Ehsan Shahrestanaki**: Formal analysis; writing—original draft; writing—review and editing. **Nooshin Ghavidel**: Conceptualization; writing—original draft; writing—review and editing; project administration; methodology.

### PEER REVIEW

The peer review history for this article is available at https://publons.com/publon/10.1002/brb3.70031.

## Data Availability

The datasets used during the current study available from the corresponding author on reasonable request.

## References

[brb370031-bib-0001] Alijanzadeh, M. , Bahrami, N. , Jafari, E. , Noori, M. , Miri, F. , Joftyar, M. , Griffiths, M. D. , & Alimoradi, Z. (2023). Iranian women's attitude toward childbearing and its' association with generalized trust, social support, marital satisfaction and governmental childbearing incentives. Heliyon, 9(5), e16162. 10.1016/j.heliyon.2023.e16162 37215895 PMC10199260

[brb370031-bib-0002] Alwan, S. , & Sadovnick, A. D. (2012). Multiple sclerosis and pregnancy: Maternal considerations. Women's Health, 8(4), 399–414. 10.2217/whe.12.33 22757731

[brb370031-bib-0003] Alwan, S. , Yee, I. M. , Dybalski, M. , Guimond, C. , Dwosh, E. , Greenwood, T. M. , Butler, R. , & Sadovnick, A. D. (2013). Reproductive decision making after the diagnosis of multiple sclerosis (MS). Multiple Sclerosis (Houndmills, Basingstoke, England), 19(3), 351–358. 10.1177/1352458512452920 22760102

[brb370031-bib-0004] Araban, M. , Karimy, M. , Armoon, B. , & Zamani‐Alavijeh, F. (2020). Factors related to childbearing intentions among women: A cross‐sectional study in health centers, Saveh, Iran. Journal of the Egyptian Public Health Association, 95(1), 1–8. 32813137 10.1186/s42506-020-0035-4 PMC7364678

[brb370031-bib-0005] Arneth, B. M. (2022). Pregnancy in patients with multiple sclerosis. Journal of Investigative Medicine, 70(1), 14–19. 10.1136/jim-2020-001609 34385291

[brb370031-bib-0006] Asghari, A. , Saed, F. , & Dibajnia, P. (2008). Psychometric properties of the depression anxiety stress scales‐21 (DASS‐21) in a non‐clinical Iranian sample. International Journal of Psychology, 2(2), 82–102.

[brb370031-bib-0007] Azami, M. , YektaKooshali, M. H. , Shohani, M. , Khorshidi, A. , & Mahmudi, L. (2019). Epidemiology of multiple sclerosis in Iran: A systematic review and meta‐analysis. PLoS ONE, 14(4), e0214738. 10.1371/journal.pone.0214738 30964886 PMC6456231

[brb370031-bib-0008] Baezzat, F. , Ahmadi Ghozlojeh, A. , Marzbani, Y. , Karimi, A. , & Azarnioshan, B. (2017). A study of psychometric properties of Persian version of attitudes toward fertility and childbearing scale. Nursing and Midwifery Journal, 15(1), 37–47.

[brb370031-bib-0009] Boeschoten, R. E. , Braamse, A. M. , Beekman, A. T. , Cuijpers, P. , van Oppen, P. , Dekker, J. , & Uitdehaag, B. M. (2017). Prevalence of depression and anxiety in multiple sclerosis: A systematic review and meta‐analysis. Journal of the Neurological Sciences, 372, 331–341. 10.1016/j.jns.2016.11.067 28017241

[brb370031-bib-0010] Bove, R. , Secor, E. , Healy, B. C. , Musallam, A. , Vaughan, T. , Glanz, B. I. , Greeke, E. , Weiner, H. L. , Chitnis, T. , Wicks, P. , & De Jager, P. L. (2013). Evaluation of an online platform for multiple sclerosis research: Patient description, validation of severity scale, and exploration of BMI effects on disease course. PloS ONE, 8(3), e59707. 10.1371/journal.pone.0059707 23527256 PMC3603866

[brb370031-bib-0011] Chen, S.‐L. , Jones, L. K. , & Jackson, M. (2018). Childbearing and quality of life decisions for women in Taiwan. International Journal of Healthcare, 4(1), 16–24. 10.5430/ijh.v4n1p16

[brb370031-bib-0012] Eslami, A. , Mahmoudi, A. , Khabiri, M. , & Najafiyan Razavi, S. M. (2014). The role of socioeconomic conditions in the citizens' motivation for participating in public sports. Applied Research in Sport Management, 2(3), 89–104.

[brb370031-bib-0013] Etemadifar, M. , Sajjadi, S. , Nasr, Z. , Firoozeei, T. S. , Abtahi, S.‐H. , Akbari, M. , & Fereidan‐Esfahani, M. (2013). Epidemiology of multiple sclerosis in Iran: A systematic review. European Neurology, 70(5–6), 356–363. 10.1159/000355140 24192707

[brb370031-bib-0014] Fattahi, N. , Saeedi Moghaddam, S. , Mohebi, F. , Rezaei, N. , Masinaei, M. , Fateh, S. M. , Soleymani Hassanlouei, E. , Manoochehri, F. , Fattahi, E. , Sahraian, M. A. , Moradi‐Lakeh, M. , Mokdad, A. H. , Naghavi, M. , & Farzadfar, F. (2021). Burden of multiple sclerosis in Iran from 1990 to 2017. BMC Neurology, 21(1), 400. 10.1186/s12883-021-02431-1 34654397 PMC8518301

[brb370031-bib-0015] Fragkoudi, A. , Rumbold, A. R. , & Grzeskowiak, L. E. (2023). Family planning and multiple sclerosis: A qualitative study of patient experiences to understand information needs and promote informed decision‐making. Patient Education and Counseling, 110, 107673. 10.1016/j.pec.2023.107673 36812770

[brb370031-bib-0016] Ghafoori, F. , Dehghan‐Nayeri, N. , Khakbazan, Z. , Hedayatnejad, M. , & Nabavi, S. M. (2020). Pregnancy and motherhood concerns surrounding women with multiple sclerosis: A qualitative content analysis. International Journal of Community Based Nursing and Midwifery, 8(1), 2–11. 32039275 10.30476/IJCBNM.2019.73900.0 PMC6969949

[brb370031-bib-0017] Hanna, M. , & Strober, L. B. (2020). Anxiety and depression in multiple sclerosis (MS): Antecedents, consequences, and differential impact on well‐being and quality of life. Multiple Sclerosis and Related Disorders, 44, 102261. 10.1016/j.msard.2020.102261 32585615 PMC7719086

[brb370031-bib-0018] Jesus‐Ribeiro, J. , Correia, I. , Martins, A. I. , Fonseca, M. , Marques, I. , Batista, S. , Nunes, C. , Macário, C. , Almeida, M. C. , & Sousa, L. (2017). Pregnancy in multiple sclerosis: A Portuguese cohort study. Multiple Sclerosis and Related Disorders, 17, 63–68. 10.1016/j.msard.2017.07.002 29055477

[brb370031-bib-0019] Kelly, E. E. , Engel, C. , Pearsall, R. , Brenton, J. N. , Bove, R. , Oh, U. , & Goldman, M. D. (2024). Multiple sclerosis and family planning: A survey study of the patient experience. Neurology: Clinical Practice, 14(1), e200222. 38148835 10.1212/CPJ.0000000000200222 PMC10751018

[brb370031-bib-0020] Kosmala‐Anderson, J. , & Wallace, L. M. (2013). A qualitative study of the childbearing experience of women living with multiple sclerosis. Disability and Rehabilitation, 35(12), 976–981. 10.3109/09638288.2012.717581 23072278

[brb370031-bib-0021] Lemus, H. N. , Warrington, A. E. , & Rodriguez, M. (2018). Multiple sclerosis: Mechanisms of disease and strategies for myelin and axonal repair. Neurologic Clinics, 36(1), 1–11. 10.1016/j.ncl.2017.08.002 29157392 PMC7125639

[brb370031-bib-0022] Lovibond, S. , & Lovibond, P. (1995). Manual for the depression anxiety stress scales Sydney . Psychological Foundation of Australia Inc.

[brb370031-bib-0023] McDonald, W. I. , Compston, A. , Edan, G. , Goodkin, D. , Hartung, H. P. , Lublin, F. D. , McFarland, H. F. , Paty, D. W. , Polman, C. H. , Reingold, S. C. , Sandberg‐Wollheim, M. , Sibley, W. , Thompson, A. , van den Noort, S. , Weinshenker, B. Y. , & Wolinsky, J. S. (2001). Recommended diagnostic criteria for multiple sclerosis: Guidelines from the international panel on the diagnosis of multiple sclerosis. Annals of Neurology: Official Journal of the American Neurological Association and the Child Neurology Society, 50(1), 121–127. 10.1002/ana.1032 11456302

[brb370031-bib-0024] Mohammadzadeh, J. , Sayehmiri, K. , & Mahmoudi, B. (2016). Standardization of social support scale (MOS) of adults who have chronic diseases in Ilam, 2015. Journal of Ilam University of Medical Sciences, 23, 69–77.

[brb370031-bib-0025] Moradi, A. , Azimi, N. , & Baharangiz, L. (2021). Recognizing the relationship between quality of life and social economic status with attitude towards having children: The case study of women in Sarpol Zahab city. Women and Society Quarterly, 11(44), 217–236.

[brb370031-bib-0026] Papa, A. , Koutelekos, I. , Stefanidou, S. , Chrysovitsanou, C. , & Polikandrioti, M. (2021). Factors associated with perceived social support of patients with multiple sclerosis. Current Journal of Neurology, 20(2), 64. 38011438 10.18502/cjn.v20i2.6741 PMC8743177

[brb370031-bib-0027] Rahmati, S. , Galavi, Z. , Kavyani, B. , Arshadi, H. , Geerts, J. , & Sharifi, H. (2024). Maternal and neonatal outcomes in pregnant women with multiple sclerosis disease: A systematic review and meta‐analysis. Midwifery, 134, 104004. 10.1016/j.midw.2024.104004 38703425

[brb370031-bib-0028] Rosati, G. (2001). The prevalence of multiple sclerosis in the world: An update. Neurological Sciences, 22(2), 117–139. 10.1007/s100720170011 11603614

[brb370031-bib-0029] Sadeghi, H. , & Saraie, H. (2016). Effective factors on mothers’ inclination to have children in Tehran Hannaneh Sadat Sadeghi, Hasan Saraie. Social Development & Welfare Planning, 7(27), 1–32.

[brb370031-bib-0030] Sherbourne, C. D. , & Stewart, A. L. (1991). The MOS social support survey. Social Science & Medicine, 32(6), 705–714. 2035047 10.1016/0277-9536(91)90150-b

[brb370031-bib-0031] Silveira, C. , Guedes, R. , Maia, D. , Curral, R. , & Coelho, R. (2019). Neuropsychiatric symptoms of multiple sclerosis: State of the art. Psychiatry Investigation, 16(12), 877. 10.30773/pi.2019.0106 31805761 PMC6933139

[brb370031-bib-0032] Söderberg, M. , Lundgren, I. , Christensson, K. , & Hildingsson, I. (2013). Attitudes toward fertility and childbearing scale: An assessment of a new instrument for women who are not yet mothers in Sweden. BMC Pregnancy and Childbirth, 13(1), 1–8. 10.1186/1471-2393-13-197 24165014 PMC4231394

[brb370031-bib-0033] Toscano, S. , Chisari, C. G. , Meli, A. , Finocchiaro, C. , Fermo, S. L. , Zappia, M. , & Patti, F. (2023). Pregnancy planning and management for women with multiple sclerosis: What has changed over the last 15 years? An Italian single‐center experience. Multiple Sclerosis and Related Disorders, 70, 104526. 10.1016/j.msard.2023.104526 36689891

[brb370031-bib-0034] Vorobeychik, G. , Black, D. , Cooper, P. , & Cox, A. (2020). Multiple sclerosis and related challenges to young women's health: Canadian expert review. Neurodegenerative Disease Management, 10(2s), 1–13. 10.2217/nmt-2020-0010 32372725

[brb370031-bib-0035] Vukusic, S. , Michel, L. , Leguy, S. , & Lebrun‐Frenay, C. (2021). Pregnancy with multiple sclerosis. Revue Neurologique, 177(3), 180–194. 10.1016/j.neurol.2020.05.005 32736812

